# Hypervariable-Locus Melting Typing: a Novel Approach for More Effective High-Resolution Melting-Based Typing, Suitable for Large Microbiological Surveillance Programs

**DOI:** 10.1128/spectrum.01009-22

**Published:** 2022-08-01

**Authors:** Matteo Perini, Aurora Piazza, Simona Panelli, Stella Papaleo, Alessandro Alvaro, Francesca Vailati, Marta Corbella, Francesca Saluzzo, Floriana Gona, Daniele Castelli, Claudio Farina, Piero Marone, Daniela Maria Cirillo, Annalisa Cavallero, Gian Vincenzo Zuccotti, Francesco Comandatore

**Affiliations:** a Department of Biomedical and Clinical Sciences, Romeo and Enrica Invernizzi Pediatric Clinical Research Center, Università Di Milano, Milan, Italy; b Department of Clinical, Surgical, Diagnostic and Pediatric Sciences, University of Pavia, Pavia, Italy; c Microbiology Institute, ASST Papa Giovanni XXIII, Bergamo, Italy; d Microbiology and Virology Unit, Fondazione IRCCS Policlinico San Matteo, Pavia, Italy; e Emerging Bacterial Pathogens Unit, Division of Immunology, Transplantation and Infectious Diseases, IRCCS San Raffaele Scientific Institute, Milan, Italy; f Laboratory Microbiology and Virology, Ospedale San Raffaele Dibit, Milan, Italy; g Laboratory of Microbiology, ASST Monza, San Gerardo Hospital, Monza, Italy; h Department of Pediatrics, Children’s Hospital Vittore Buzzi, Università Di Milano, Milan, Italy; ATCC

**Keywords:** microbiological surveillance, high-resolution melting, outbreak reconstruction, low- and middle-income countries, real-time surveillance

## Abstract

Pathogen typing is pivotal to detecting the emergence of high-risk clones in hospital settings and to limit their spread. Unfortunately, the most commonly used typing methods (i.e., pulsed-field gel electrophoresis [PFGE], multilocus sequence typing [MLST], and whole-genome sequencing [WGS]) are expensive or time-consuming, limiting their application to real-time surveillance. High-resolution melting (HRM) can be applied to perform cost-effective and fast pathogen typing, but developing highly discriminatory protocols is challenging. Here, we present hypervariable-locus melting typing (HLMT), a novel approach to HRM-based typing that enables the development of more effective and portable typing protocols. HLMT types the strains by assigning them to melting types (MTs) on the basis of a reference data set (HLMT-assignment) and/or by clustering them using melting temperatures (HLMT-clustering). We applied the HLMT protocol developed on the capsular gene *wzi* for Klebsiella pneumoniae on 134 strains collected during surveillance programs in four hospitals. Then, we compared the HLMT results to those obtained using *wzi*, MLST, WGS, and PFGE typing. HLMT distinguished most of the K. pneumoniae high-risk clones with a sensitivity comparable to that of PFGE and MLST+*wzi*. It also drew surveillance epidemiological curves comparable to those obtained using MLST+*wzi*, PFGE, and WGS typing. Furthermore, the results obtained using HLMT-assignment were consistent with those of *wzi* typing for 95% of the typed strains, with a Jaccard index value of 0.9. HLMT is a fast and scalable approach for pathogen typing, suitable for real-time hospital microbiological surveillance. HLMT is also inexpensive, and thus, it is applicable for infection control programs in low- and middle-income countries.

**IMPORTANCE** In this work, we describe hypervariable-locus melting typing (HLMT), a novel fast approach to pathogen typing using the high-resolution melting (HRM) assay. The method includes a novel approach for gene target selection, primer design, and HRM data analysis. We successfully applied this method to distinguish the high-risk clones of Klebsiella pneumoniae, one of the most important nosocomial pathogens worldwide. We also compared HLMT to typing using WGS, the capsular gene *wzi*, MLST, and PFGE. Our results show that HLMT is a typing method suitable for real-time epidemiological investigation. The application of HLMT to hospital microbiology surveillance can help to rapidly detect outbreak emergence, improving the effectiveness of infection control strategies.

## INTRODUCTION

Healthcare-associated infections (HAIs) are a major burden for global public health (1). Microbiological surveillance programs are pivotal to establishing effective infection control strategies. In particular, subspecies typing is fundamental for detecting the emergence of high-risk clones. The most commonly used methods for subspecies bacterial typing are pulsed-field gel electrophoresis (PFGE), multilocus sequence typing (MLST), and whole-genome sequencing (WGS). All these methods require several hours (up to days) to be performed, and this limits their application for real-time nosocomial surveillance programs.

The high-resolution melting (HRM) assay has been proposed as a suitable method for fast bacterial typing (2, 3). This technique measures the melting temperatures of quantitative PCR (qPCR) amplicons, which depend on the GC content. HRM can even distinguish amplicons diverging by just one single nucleotide polymorphism (SNP), and it is widely used to detect human allele variants (4). HRM protocols designed on hypervariable genes are able to discriminate among bacterial clones within the same species (2, 3), because their amplicons will melt at different temperatures (e.g., they differ in GC content). HRM is particularly promising for microbiological surveillance: it is fast (~5 h to complete the analysis), discriminatory, and inexpensive (~5 euros per sample), and it can be performed on the most common qPCR platforms (5).

Despite the numerous HRM protocols proposed so far for bacterial typing (2), the method is rarely applied in hospital settings for microbiological surveillance. Indeed, most protocols have been designed to distinguish only between the few clones used for protocol development, including only a fraction of the entire genetic variability of the pathogen. Moreover, only a few algorithms and software programs are available to analyze HRM data for epidemiological purposes.

Here, we describe the validation of a proof of concept for HRM-based subspecies typing. We focused on hypervariable genes and implemented a tool (i.e., EasyPrimer) to facilitate HRM primer design in this difficult context (3). Additionally, we developed an algorithm for pathogen typing using HRM data (6). We already used this approach to develop an HRM-based typing protocol for Klebsiella pneumoniae on the capsular gene *wzi* (3), and then we validated the repeatability and portability of the method (5).

In this study, we provide a comprehensive description of this novel approach, which we named hypervariable-locus melting typing (HLMT). We applied HLMT in four nosocomial epidemiological investigations on Klebsiella pneumoniae, comparing its typing efficiency to that of *wzi*, MLST, WGS, and PFGE.

## RESULTS

### Typing method results.

Strains from data sets from four Italian hospitals (San Gerardo Hospital in Monza [HSG], IRCCS Fondazione Policlinico San Matteo Hospital in Pavia [PSM], ASST Papa Giovanni XXIII Hospital in Bergamo [PG23], and IRCCS San Raffaele Hospital in Milan [OSR]) were typed using hypervariable locus melting typing (HLMT-assigment and HLMT-clustering), MLST, *wzi*, and WGS. In addition, the PFGE typing results of the 80 strains in the OSR data set were retrieved from Gona and colleagues (7). The results of HLMT, MLST, *wzi*, WGS, and PFGE typing are reported in Table S1 in the supplemental material, and the core SNP-based phylogenetic trees used for WGS typing are shown in Fig. S1.

### Comparison of the typing methods.

**FIG 1:**
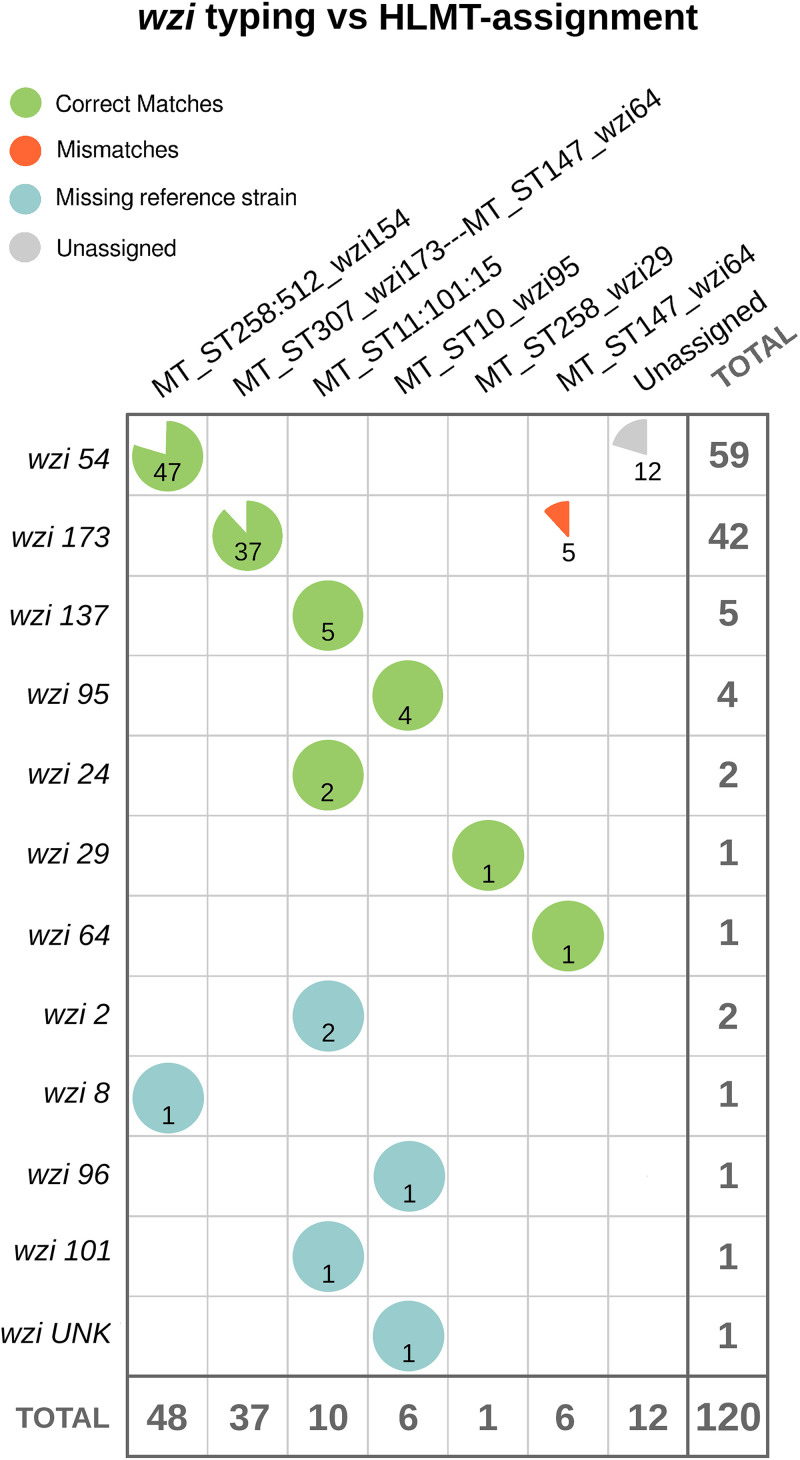
Correlation matrix between *wzi* allele typing and hypervariable-locus melting typing (HLMT)-assignment for 120 Klebsiella pneumoniae strains spanning 12 *wzi* alleles. The pie charts indicate, for each *wzi* allele, the proportion of matches with the melting types (MT). The green pies show correct matches and the red ones the mismatches. The light blue pies indicate the strains belonging to *wzi* alleles absent from the reference data set used to perform the HLMT-assignment analysis. The gray pies indicate strains that were classified as “unassigned” by the HLMT-assignment analysis.

The HLMT-assignment algorithm assigns the strains into the melting types (MTs) present in the reference database. This analysis was able to assign 120 of the 134 strains (~90%) included in this study. Of these 120 strains, 6 (5%) belonged to *wzi* alleles not included in the HLMT reference strain data set used for the analyses (see Materials and Methods). This made it impossible to assess, for these six strains, if the HLMT-assignment and the *wzi* allele were coherent. Nine strains were not included in the evaluation to avoid the overestimation of the metrics, because they were part of the reference data set (see Materials and Methods). For 102 of the remaining 107 strains (95%), the HLMT-assignment and the *wzi* allele were coherent, with a Jaccard similarity index value of 0.9. The HLMT-assignment results are reported in Table S1, and the correlation matrix plot of the HLMT-assignment versus *wzi* is shown in Fig. 1.

**FIG 2:**
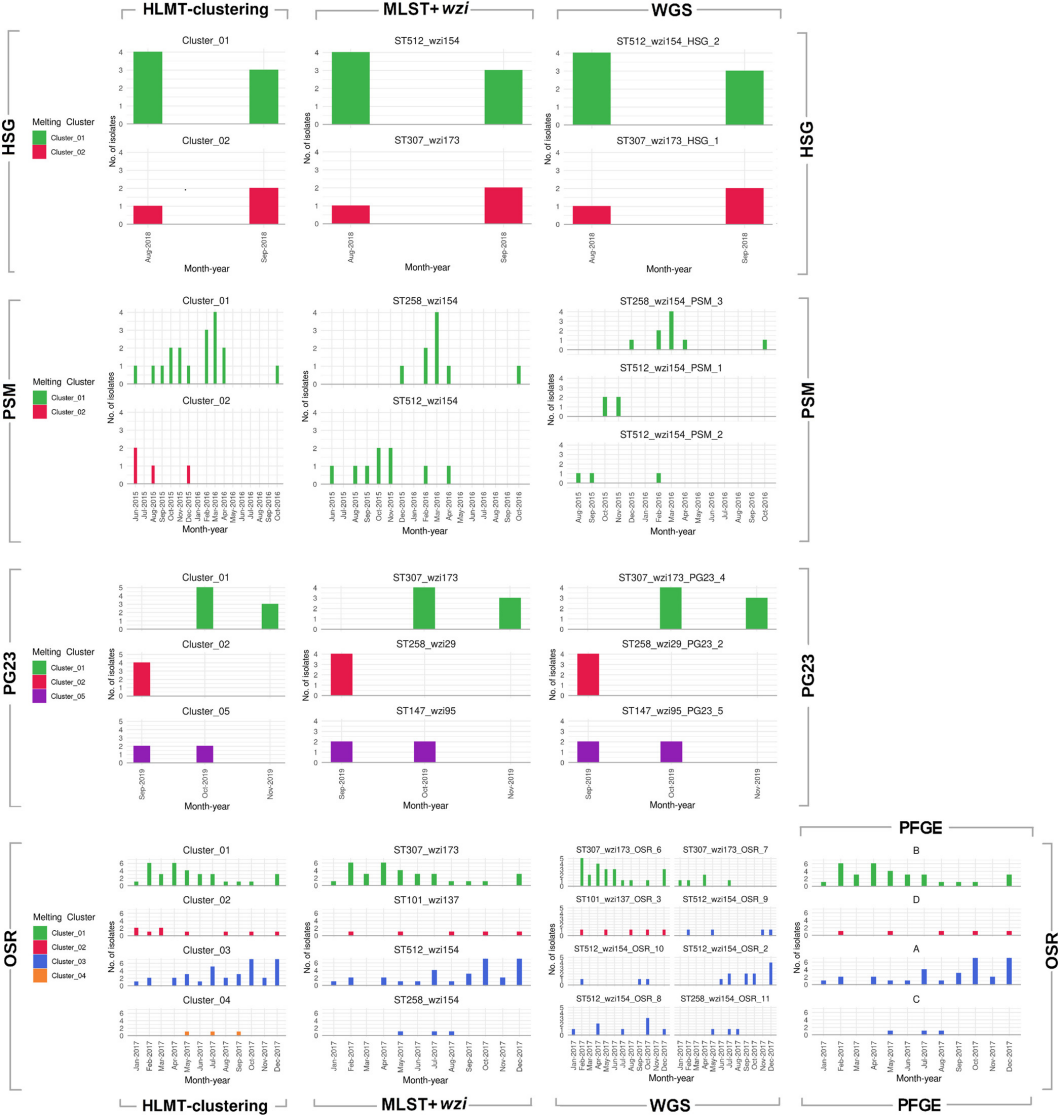
Epidemiological curves reconstructed using the isolation dates and typing information obtained by HLMT-clustering, MLST+*wzi*, WGS, and PFGE from the four data sets analyzed in this study. Each row of bar plots refers to a data set and each column to a typing method. The colors used in the bar plots correspond to the HLMT clusters.

For each data set, the epidemiological curves obtained combining the typing information (HLMT-clustering, MLST profile in combination with wzi gene allele [MLST+*wzi*], see Material and Methods, WGS, and PFGE) and isolation dates of the strains are shown in Fig. 2. In addition, the HLMT-clustering results were compared to those obtained using MLST+*wzi* and WGS; graphical representations of the relative contingency tables are provided in Fig. S2 and S3, respectively. The HLMT-clustering results were compared to those for PFGE for the OSR data set only (see Fig. S4).

## DISCUSSION

Genetic studies show that for several bacterial pathogens, most nosocomial infections are caused by a very limited number of specific lineages, called high-risk clones (8–12). For instance, most of the multidrug-resistant K. pneumoniae strains isolated from hospital patients worldwide belong to a few sequence types (STs; ST258, ST307, ST11, ST147, and ST15) among the over 6,000 STs already described in BIGSdb (13). Thus, the rapid identification and discrimination of high-risk clones is pivotal to establishing effective infection control strategies. The high-resolution melting (HRM) assay can be used to rapidly distinguish pathogen clones on the basis of the melting temperature of the target genes. HRM-based typing requires a few hours per sample at a cost of a few euros, and it is particularly suitable when several strains have to be typed, such as in large screenings and surveillance programs. Unfortunately, designing highly discriminatory HRM protocols and data analysis can be challenging.

**FIG 3:**
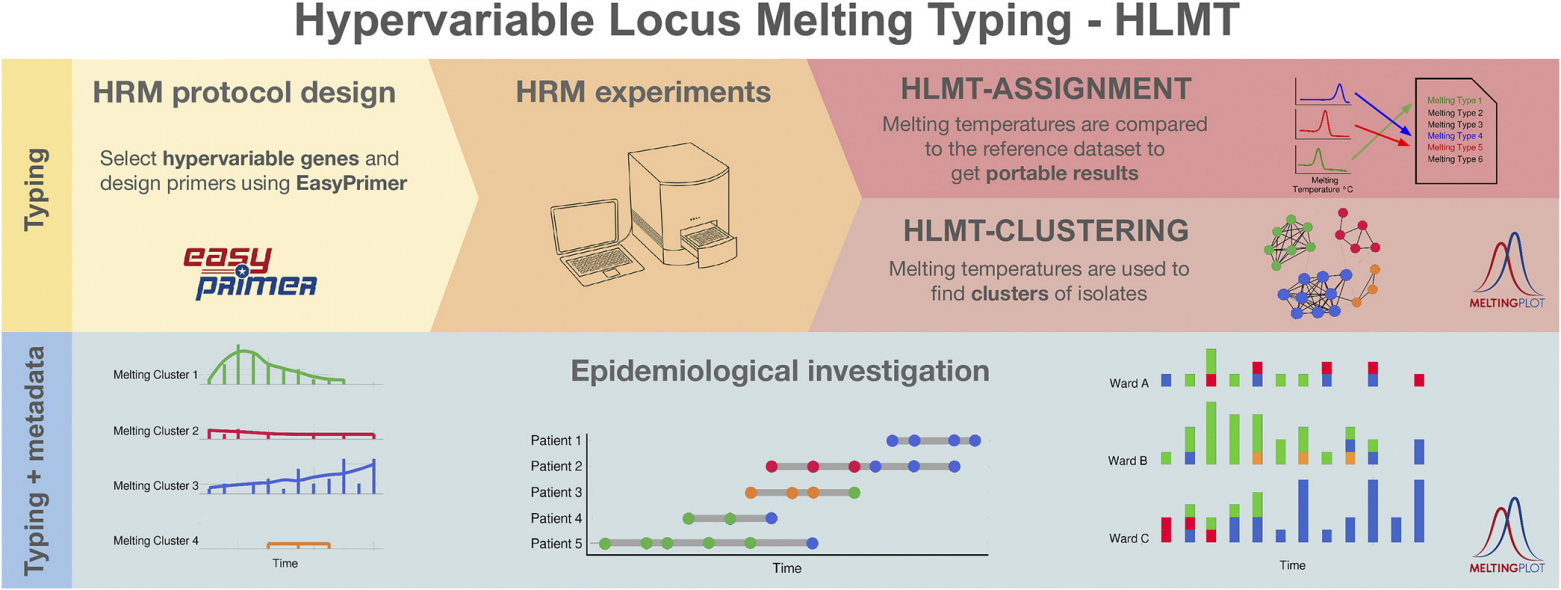
Graphical representation of the flow of hypervariable-locus melting typing (HLMT). (Top) The three main steps of the HLMT method: HRM protocol design on hypervariable genes, HRM experiments, and HRM data analysis for typing (HLMT-clustering and HLMT-assignment). (Bottom) The combination of HLMT typing and isolate metadata enables the performance of epidemiological investigations, e.g., the construction of epidemiological curves and patient timelines. Primer design, HLMT-clustering, HLMT-assignment, and epidemiological curve production can be performed using free online user-friendly tools (namely, EasyPrimer for primer design and MeltingPlot v2.0 for the other tasks).

With this proof-of-concept study, we show the nosocomial applicability of hypervariable-locus melting typing (HLMT), an innovative approach to high-resolution melting (HRM)-based typing. HLMT makes it easier to design highly discriminatory HRM protocols and to perform reliable, robust, repeatable, and portable HRM-based typing analyses. We applied HLMT to the typing of Klebsiella pneumoniae in four real hospital scenarios, comparing the results with those obtained by more established approaches, whole-genome sequencing (WGS), multilocus sequence typing (MLST), pulsed-field gel electrophoresis (PFGE), and *wzi* typing. As summarized in Fig. 3, the HLMT workflow consists of three main parts: (i) HLMT protocol design on hypervariable genes; (ii) HRM experiments; and (iii) HRM data analysis for HLMT-assignment and HLMT-clustering.

### HRM primer design.

HRM is less sensitive than sequencing for discriminating among DNA sequences. Thus, to obtain a stronger signal, the HRM protocol should include more than one primer pair, and it should be designed on hypervariable genes. Primer design on hypervariable genes is challenging because it requires the analysis of up to thousands of different gene alleles to identify conserved regions suitable for primer design. The identification of these gene regions can be easily performed using the online tool EasyPrimer (3). With this tool, multiple primer sets can be designed on the same or different hypervariable genes, increasing the discrimination power of the HLMT protocol.

### HLMT-assignment and HLMT-clustering.

HLMT analysis includes two different strain typing methods: HLMT-assignment and HLMT-clustering. HLMT-assignment analysis assigns each strain to a melting type (MT) by comparing its melting temperatures to a reference data set. The data set consists of the melting temperatures of previously typed strains, selected to represent the genetic variability of the pathogen (see Materials and Methods). On the other hand, HLMT-clustering groups strains on the basis of their melting temperatures using a graph-based clustering algorithm (6). Strains with similar melting temperatures for all the primers included in the HLMT protocol are grouped together. This clustering approach recalls PFGE clustering, where a hierarchical clustering algorithm is used to group the strains on the basis of their restriction patterns.

The strength of HLMT-assignment over HLMT-clustering is that the HLMT-assignment results are portable; they can be shared among laboratories and/or compared to previous HLMT experiments performed on the same model of real-time PCR instrument (5). On the other hand, HLMT-clustering works even on strains belonging to lineages absent from the HLMT strain data set.

The discrimination power described in this work was obtained using just two primer pairs designed on a single gene. The use of additional primer pairs would increase considerably the discrimination power, but it would also make data analysis more complex. To tackle this issue, we developed the tool MeltingPlot v2.0 (6), which automatically performs HLMT-assignment and HLMT-clustering analyses using HRM data regardless of the number of primers that make up the HLMT protocol.

### HLMT protocol for K. pneumoniae.

We previously designed an HLMT protocol for K. pneumoniae typing (3). In this work, we show the applicability of the method for hospital surveillance programs. We followed the three steps of the workflow described in Fig. 3: (i) HRM protocol design was previously carried out by Perini et al. (3), selecting the hypervariable capsular gene *wzi* as the target and designing two primer sets (wzi-3 and wzi-4) by analyzing the hundreds of *wzi* allele sequences available, using the EasyPrimer tool; (ii) HRM experiments were performed in this work; (iii) HLMT-assignment was performed using the HLMT reference strain data set (see Materials and Methods), and HLMT-clustering analyses were performed using the melting temperatures of the 134 strains in the four data sets, using the MeltingPlot v2.0 tool (6).

We reconstructed the K. pneumoniae reference data set, naming the melting types with the sequence types and *wzi* alleles of the most epidemiologically relevant clone(s) present in the MT, recalling the labeling approach used in MLST for clonal complexes (e.g., K. pneumoniae CC258). The HLMT-assignment analysis classified almost every strain in concordance with its *wzi* allele (Fig. 1), showing the portability and repeatability power of the method.

As shown in Fig. 2, the HLMT-clustering epidemiological scenarios were highly consistent with those obtained using the other typing methods: the curves are very similar for the HSG, PG23, and OSR data sets, and HLMT-clustering was also able to detect the emergence of the outbreak at PSM.

Furthermore, although HLMT-clustering is less sensitive than MLST+*wzi*, the method was able to discriminate between the most epidemiologically relevant STs (i.e., ST258 *wzi*29, ST258 *wzi*154, ST307 *wzi*173, and ST11), and the only groups of high-risk clones not distinguishable were ST101-ST11-ST15 and ST307-ST147 (Fig. S2 in the supplemental material). Here, we also want to highlight that HLMT was able to discriminate between the two major subclones of the pandemic ST258, Clade1 (harboring *wzi*29) and Clade2 (*wzi*154). These two clones, harboring the same MLST gene alleles, were detected for the first time using WGS analysis (14), and they are not distinguishable using MLST typing without further sequencing the gene *wzi*.

HLMT is less discriminatory than WGS, but it is fast, easy, inexpensive, and also repeatable. The lower sensitivity of HRM in comparison to sequencing means that HLMT is unable to discriminate between clones harboring target genes with similar melting temperatures (e.g., ST101, ST11, and ST15, which harbor *wzi* alleles with similar melting temperatures). Nevertheless, as shown in this work, this low sensitivity does not affect the repeatability of the method; indeed, it fails to distinguish only a few specific clones. On the other hand, this low sensitivity drastically reduces the possibility of obtaining false-negative results. Therefore, when two strains are assigned to two distinct melting types (MT) or two melting clusters, we can safely exclude their genetic relatedness. Having this information quickly during the early stage of a nosocomial outbreak can be particularly useful to establish an effective infection control strategy.

### Portability.

HLMT analysis can be performed using HRM data obtained from any HRM-capable qPCR platform. Variations in salt concentration of the mix and impurities derived from DNA extraction may alter the melting temperature profiles of the same strains (2). Nevertheless, as shown by Pasala and colleagues (5), the graph-based clustering algorithm implemented in MeltingPlot (6) is able to group the strains in the same HLMT clusters even if the HRM experiments are carried out in different laboratories with the same model of instrument, without the need to add standard strains to the data set (5). Regardless, the use of standard DNA extraction procedures and reagent mixes should decrease the possibility of measuring unexpected variations of the melting temperatures.

### Applicability.

The only two instruments required to perform HLMT are an HRM-capable qPCR platform and a standard personal computer (PC); the molecular biology skills required are also minimal. No bioinformatics skills are necessary because both HLMT-assignment and HLMT-clustering can be automatically performed online, using the free and user-friendly tool MeltingPlot (6).

**FIG 4:**
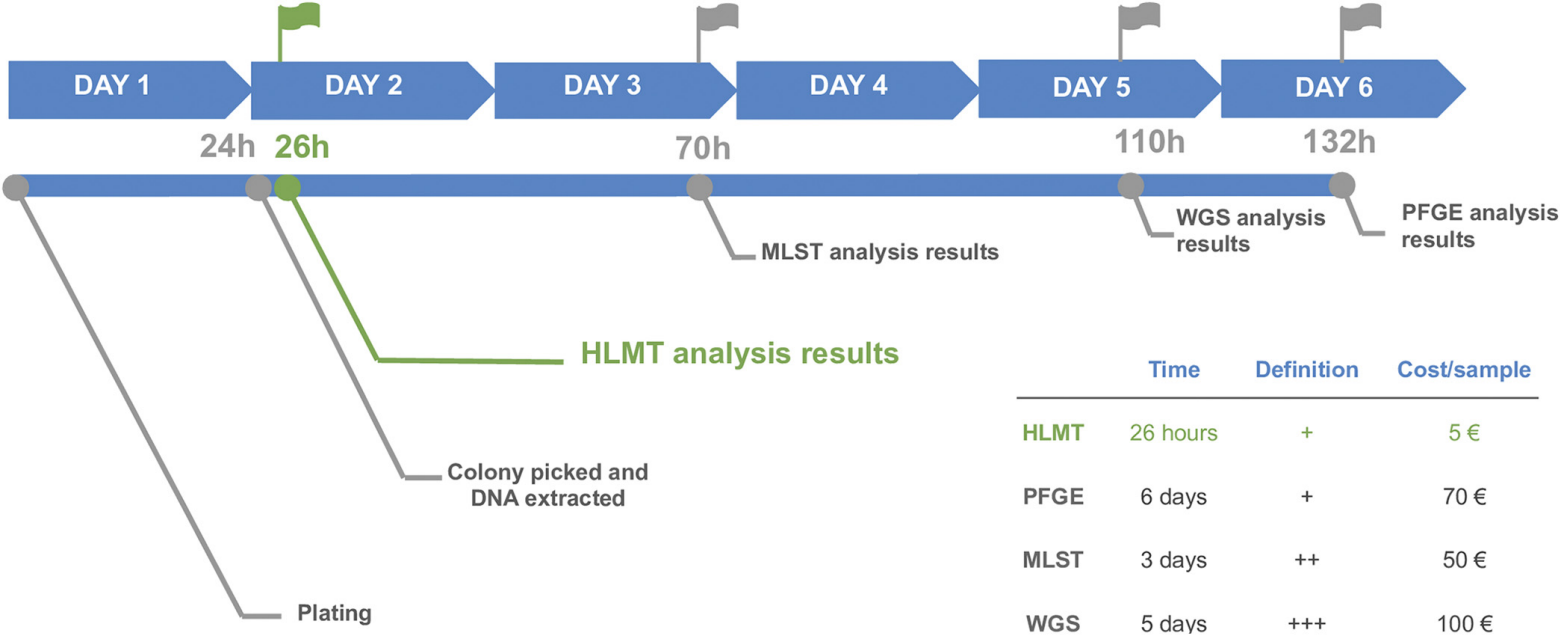
Timeline and cost of different typing methods: hypervariable-locus melting typing (HLMT) (in green), hypervariable-locus melting typing (MLST), whole-genome sequencing (WGS), and pulsed-field gel electrophoresis (PFGE). The time required to perform each typing analysis, the relative typing definition, and the cost per sample are also reported.

When the metadata of the strains are available, MeltingPlot can be used to merge HLMT cluster and strain metadata (isolation date and location) to produce graphical descriptions of the epidemiological scenario under study. Lastly, using HLMT, it is possible to type large numbers of isolates in a few hours for a few euros per sample (Fig. 4).

WGS is the most sensitive method for bacterial typing; but even as it is becoming the gold standard typing approach for several pathogen species, PFGE and MLST are applied more often in real-time hospital surveillance programs (15). In this work, we show that HLMT is four times faster than MLST and 33 times faster than PFGE, maintaining a sensitivity comparable to these typing methods. Moreover, the low cost of HLMT (~5 euros/isolate) makes it one of the most inexpensive subspecies pathogen typing methods available, up to 20 times less expensive than MLST, PFGE, or WGS (Fig. 4). This makes HLMT suitable for epidemiological investigations in real time and for the development of low-cost surveillance programs even in low- and middle-income countries.

## MATERIALS AND METHODS

### Ethics statement.

This study uses bacterial isolates from human samples that were obtained as part of hospital routines. No extra human samples were obtained for this research. Therefore, informed consent (either written or verbal) was not required.

### Isolate data sets.

The strain data sets used for this study were obtained using isolates collected during microbiological surveillance programs in four Italian hospitals. The Italian hospitals included in the study were San Gerardo Hospital in Monza (HSG), IRCCS Fondazione Policlinico San Matteo Hospital in Pavia (PSM), ASST Papa Giovanni XXIII Hospital in Bergamo (PG23), and IRCCS San Raffaele Hospital in Milan (OSR). The hospitals provided a total of 134 Klebsiella pneumoniae strains. (i) HSG provided 10 strains isolated during an outbreak that involved 10 patients in the oncohematology ward between 14 August 2018 and 24 September 2018 (HSG data set). (ii) PSM provided 24 strains isolated during an outbreak, which were previously investigated using whole-genome sequencing (WGS) and described by Ferrari and colleagues (16) (PSM data set) (the original data set consisted of 32 strains, but only 24 were successfully revitalized in this work). (iii) PG23 provided 20 strains isolated during an outbreak that involved 16 patients and nine hospital wards between 7 May 2019 and 4 November 2019 (PG23 data set). (iv) OSR provided all 80 strains isolated during a one-year-long WGS surveillance in 2017, which were previously typed using WGS and PFGE by Gona and colleagues (7) (OSR data set). Of the 134 strains, 30 were subjected to WGS in this work; 12 of the 134 strains were used by Perini and colleagues (3) for the development of the K. pneumoniae HLMT typing protocol used in this work. Details of the study strains are reported in Table S1 in the supplemental material.

### DNA extraction.

The frozen stocks were maintained at −80°C on LB medium with 40% glycerol. For each of the 134 strains, a bacterial culture was subjected to two consecutive single-colony selections on MacConkey agar, incubated overnight at 37°C (Becton, Dickinson, Franklin Lakes, NJ, USA). A single bacterial colony was then suspended in liquid medium and incubated overnight, and DNA was extracted using the DNeasy blood and tissue kit following the manufacturer’s instructions (Qiagen, Hilden, Germany).

### WGS-based typing.

**(i) Whole-genome sequencing**. Thirty isolates from the HSG and PG23 data sets were subjected to WGS on the Illumina MiSeq platform (San Diego, CA, USA), after Nextera XT 2 × 250-bp paired-end library preparation. The obtained reads were quality checked using FastQC and trimmed using Trimmomatic software (17). The trimmed reads were then assembled using SPAdes (18). The remaining 104 K. pneumoniae strains isolated from PSM (*n* = 24) and OSR (*n* = 80) were subjected to WGS in previous studies, and the authors used the same library preparation kit and the same software to check the read quality, to trim the reads, and to perform the genome assembly (7, 16).

**(ii) Genomic background data set**. The 15,699 public genome assemblies of K. pneumoniae present in the PATRIC database (19) on 8 February 2021 for which the publication code was available (in accordance with the Fort Lauderdale and Toronto agreements) were retrieved.

**(iii) Single nucleotide polymorphism-based phylogenetic reconstruction and typing**. Each of the four data sets was separately subjected to core SNP calling as follows. (i) Each genome was compared to the retrieved PATRIC data set using Mash (20), and the 50 most similar strains were included in the background data set. (ii) These selected PATRIC genomes were merged with the data set genomes. (iii) Core SNP calling was performed on the merged genome data set using the tool Purple (7). For each of the four data sets, the obtained core SNP alignment was subjected to maximum likelihood (ML) phylogenetic analysis with 100 bootstraps using the software RAxML8 (21), after selection of the best model using ModelTest-NG (22) (generalized time-reversible with gamma distribution [GTR+G] for HSG and PG23, and TransVersion model with gamma distribution [TVM+G] for the PSM and OSR data sets). For each of the four data sets, clusters were identified on the resulting trees as the largest monophylum of data set strains (not from PATRIC) with bootstrap support of ≥75.

### Multilocus sequence typing and *wzi* alleles.

K. pneumoniae clones are often defined by combining the multilocus sequence typing (MLST) (23) profile and the *wzi* gene allele (24). The MLST profiles and *wzi* alleles of the 134 genome assemblies were determined using the tool Kleborate (25).

### Pulsed-field gel electrophoresis clustering.

Pulsed-field gel electrophoresis (PFGE) clusters were described by Gona and colleagues (7) for the 80 strains of the OSR data set following digestion with the XbaI enzyme and separation into a CHEF-DR III electrophoretic system (Bio-Rad, Hercules, CA). Briefly, the PFGE clusters were identified using InfoQuest FP v5.1 software (Bio-Rad), and they were confirmed by the epidemiological scenario (7).

### Hypervariable-locus melting typing.

**(i) *Klebsiella pneumoniae* HLMT reference data set reconstruction.** The HLMT protocol used in this work was developed by Perini and colleagues (2020) on the K. pneumoniae capsular gene *wzi* using two primer sets (wzi-3 and wzi-4) (3). *Wzi* was selected as the target gene because it is genetically hypervariable, since it is subjected to diversifying selection as a consequence of immune escape (26). Furthermore, this gene is often used for typing the bacterium, given the strong correlation between the *wzi* allele and K. pneumoniae sequence type (ST) (24). We reconstructed a database of melting types (MTs) and wzi-3 and wzi-4 melting temperatures, exploiting the data set used by Pasala et al. (5) to validate the repeatability and portability of the protocol (43 K. pneumoniae strains belonging to epidemiologically relevant STs, harboring 12 different *wzi* alleles). We clustered these 43 strains on the basis of their wzi-3 and wzi-4 melting temperatures, using MeltingPlot v2.0 (5). The obtained clusters were called melting types. Then, for each MT, we computed the reference melting temperatures for the wzi-3 and wzi-4 primers as the midrange melting temperatures (the arithmetic mean of the highest and the lowest temperature) of the strains in that MT. Lastly, we labeled the MTs using the names of the most relevant lineages of the strains that they contain. The reference data set is available at https://skynet.unimi.it/wp-content/uploads/TemplateHLMT_ref_KPN_22-02-2022.xls.

**(ii) High-resolution melting experiments**. All the K. pneumoniae strains included in this work were subjected to high-resolution melting (HRM) assays using the protocol described by Perini et al. (3). The HRM experiments were performed with the two primer sets designed on the *wzi* gene proposed by Perini and colleagues (3) (wzi-3 forward, GCTTAYGCRGCYGGGTTAGTRGT; wzi-3 reverse, GGCCASGTCGACARGCTCAG; wzi-4 forward, GCCGCTRAGYCAGGAAGAGAT; wzi-4 reverse, GACTGTCWGCBTTRAAAGCSGA). The experiments were performed using the Bio-Rad CFX Connect real-time PCR system. Each experiment was performed in a 10-μL volume which contained the following: 5 μL of 2× SsoAdvanced universal SYBR Green supermix (Bio-Rad), 0.4 μL of each primer (0.4 μM), and 1 μL of template DNA (25 to 50 ng/μL). The thermal profile used for the experiment was as follows: 98°C for 2 min; 40 cycles of 95°C for 7 s, 61°C for 7 s, and 72°C for 15 s; 95°C for 2 min. The subsequent HRM analysis ranged from 70°C to 95°C, with fluorescence data acquisition every 0.5°C increment. Three technical replicates were performed for each strain and for each primer pair. Negative controls were added in every run for the two primer pairs.

**(iii) Hypervariable-locus melting typing data analysis and comparison**. For each of the four data sets (HSG, PSM, PG23, and OSR), the obtained wzi-3 and wzi-4 melting temperatures and the above-mentioned reference data set were used to perform HLMT-assignment and HLMT-clustering analyses using the MeltingPlot v2.0 tool (6) (available online at https://skynet.unimi.it/index.php/tools/MeltingPlot/). HLMT-assignment analysis is performed by comparing the melting temperatures of each study isolate to the melting temperatures of the Klebsiella pneumoniae strains of the HLMT reference data set. A study isolate is assigned to a melting type (MT) when both the wzi-3, and wzi-4 melting temperatures differ by ≤0.5°C from the wzi-3 and wzi-4 temperatures of the reference isolate of that MT, using the same temperature threshold of the HRM clustering algorithm implemented in MeltingPlot (6). Considering that this HLMT protocol was designed on the *wzi* gene, we tested the accuracy of the HLMT-assignment analysis by evaluating the correspondence between MTs and *wzi* alleles. Of the 134 study strains, 9 were the same as those used by Pasala et al. (5) (see Table S1). This data set was used to reconstruct the Klebsiella pneumoniae HLMT reference data set (see above). Thus, to avoid overestimation of the accuracy of the HLMT-assignment analysis, these nine strains were not included for computing the Jaccard similarity index (using the R package clusteval [27]), which was used to evaluate the similarity between the HLMT-assignment and *wzi* typing. In addition, a correlation plot of the HLMT-assignment against *wzi* typing was computed using the R package corrplot (28). To perform the HLMT-clustering analysis, MeltingPlot v2.0 (6) was used to define clusters of isolates with the melting temperatures measured by the HRM experiments, following the graph-based algorithm described by Perini and colleagues (6). The same algorithm was used previously to validate the HRM protocol (3) and to show its repeatability and portability (5). The HLMT-clustering results were compared to the results of PFGE, MLST+*wzi*, and WGS by means of heat maps produced using the R package gplots (29). To determine if HLMT-clustering would be a useful tool for microbiological surveillance in a real hospital scenario, the HLMT-clustering, MLST+*wzi*, and WGS typing results were combined with the collection dates of the strains to obtain epidemiological curves showing the prevalence of the clusters and groups over time (6). Additionally, PFGE typing results were retrieved from the work of Gona et al. (7) and used to obtain relative epidemiological curves.

### Data availability.

The genome assemblies for the 30 K. pneumoniae strains (10 from the HSG data set and 20 from the PG23 data set) sequenced in this work are available at the NCBI repository under BioProject accession number PRJEB44864 (SRA accession number ERP128959). The assembly statistics and accession numbers are reported in Table S1 in the supplemental material.
