# Supplementary material for: Hypervariable-Locus Melting Typing: a Novel Approach for More Effective High-Resolution Melting-Based Typing, Suitable for Large Microbiological Surveillance Programs
Source: Microbiol Spectr. 2022 Aug 1;10(4):e01009-22. doi: 10.1128/spectrum.01009-22 (PMC9430602; doi:10.1128/spectrum.01009-22)
Supplement: Supplemental file 1 — Supplemental material. Download spectrum.01009-22-s0001.pdf, PDF file, 1.9 MB [file spectrum.01009-22-s0001.pdf]

FigureS1: WGS phylogenetic analysis - Maximum Likelihood phylogenetic trees of the four datasets used in this study (HSG, PSM, PG23, OSR). Each dataset was analyzed with the closest genomes found in PATRIC online database. The WGS cluster of each genome used in this study is shown by the colors next to the strain name.

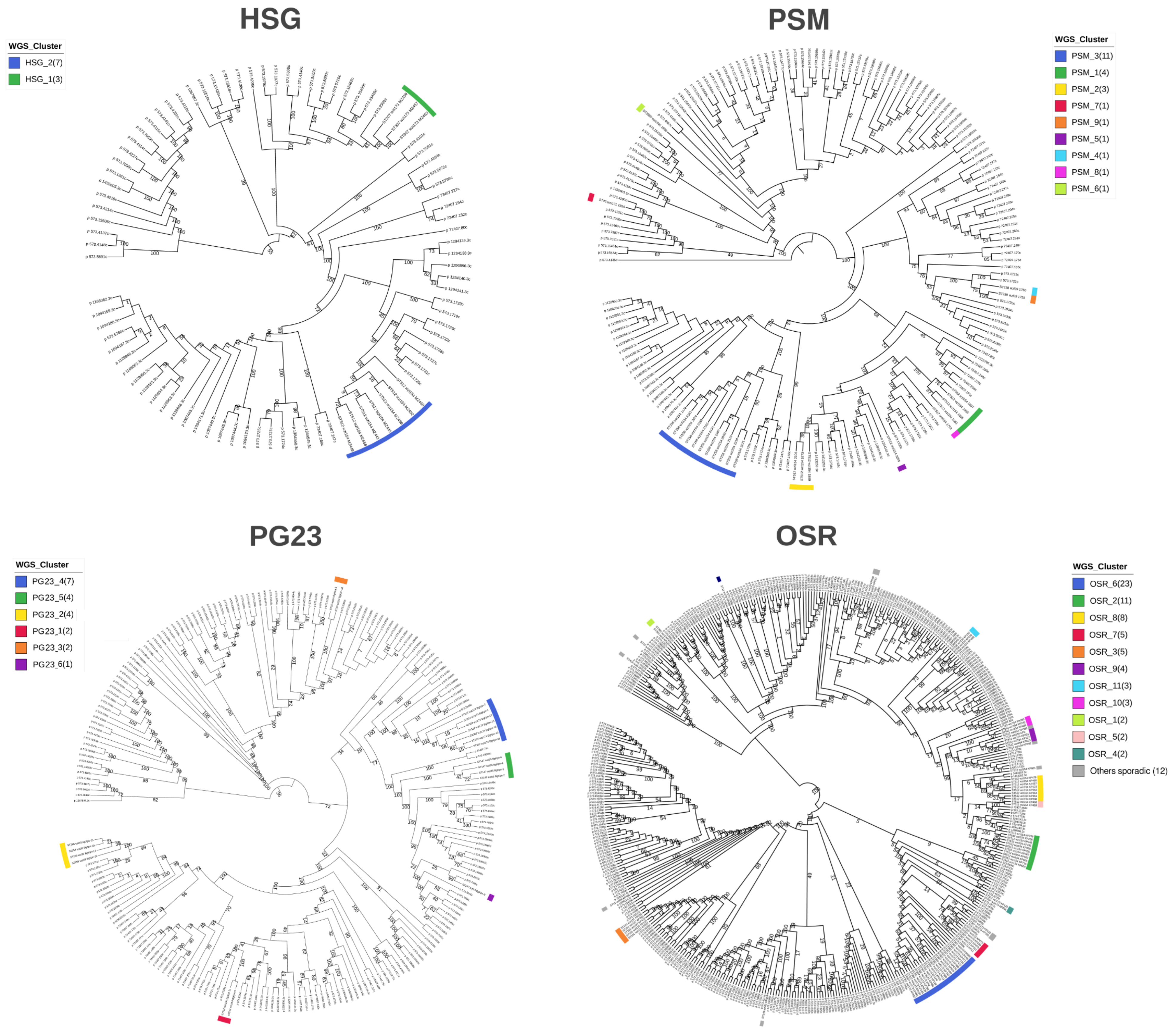

FigureS2: Contingency tables of HLMT-clustering vs MLST+*wzi* - For each of the four datasets analyzed in this study (HSG, PSM, PG23, OSR), the HLMT clusters are compared to the MLST+*wzi* groups with a contingency table. Each color represents a HLMT cluster. The gray color indicates the strains that the HLMT clustering algorithm classified as “Undetermined”.

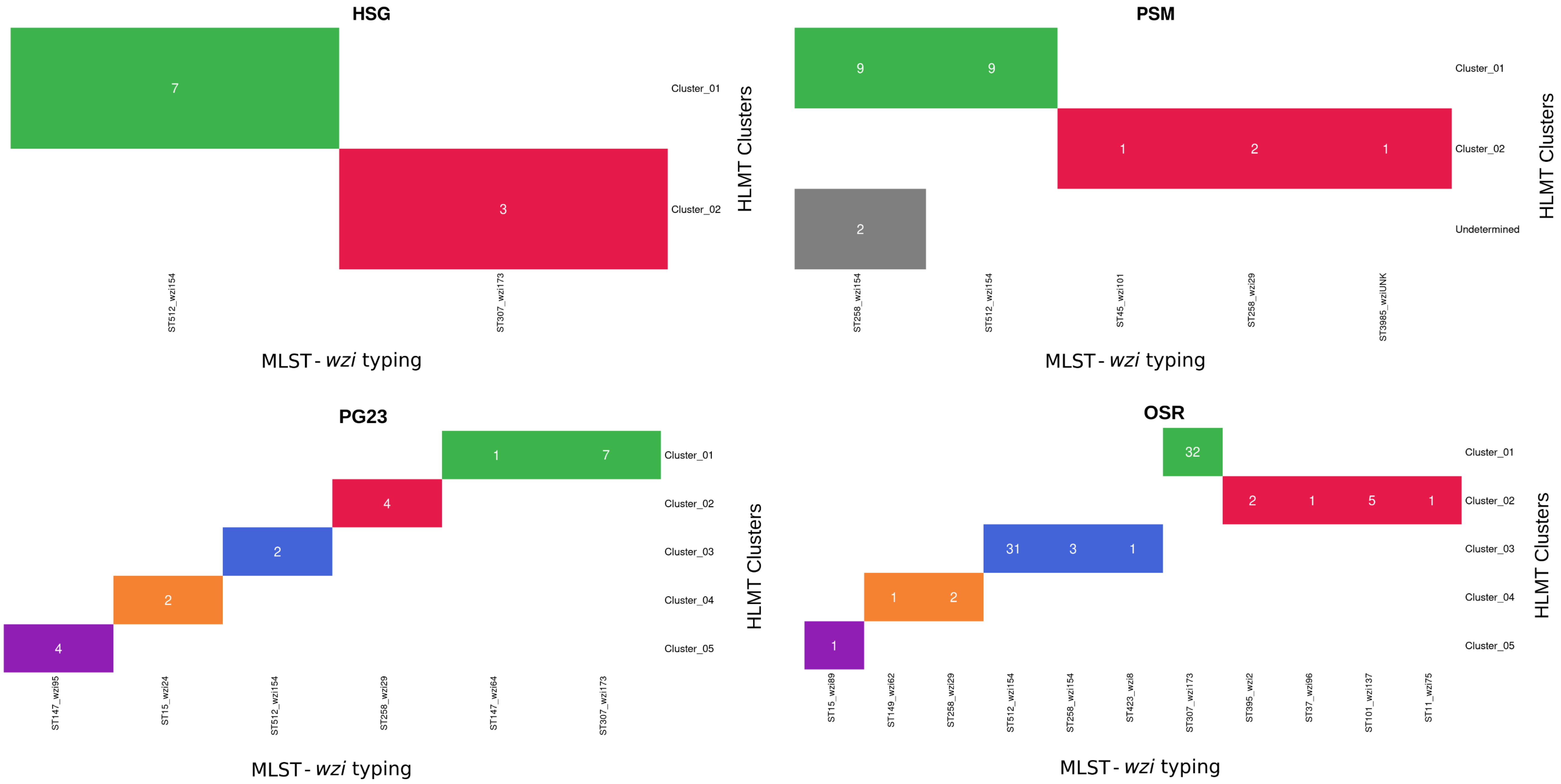

FigureS3: Contingency tables of HLMT-clustering vs WGS - For each of the four datasets analyzed in this study (HSG, PSM, PG23, OSR), the HLMT clusters are compared to the WGS clusters with a contingency table. Each color represents a HLMT cluster. The gray color indicates the strains that the HLMT clustering algorithm classifies as “Undetermined”.

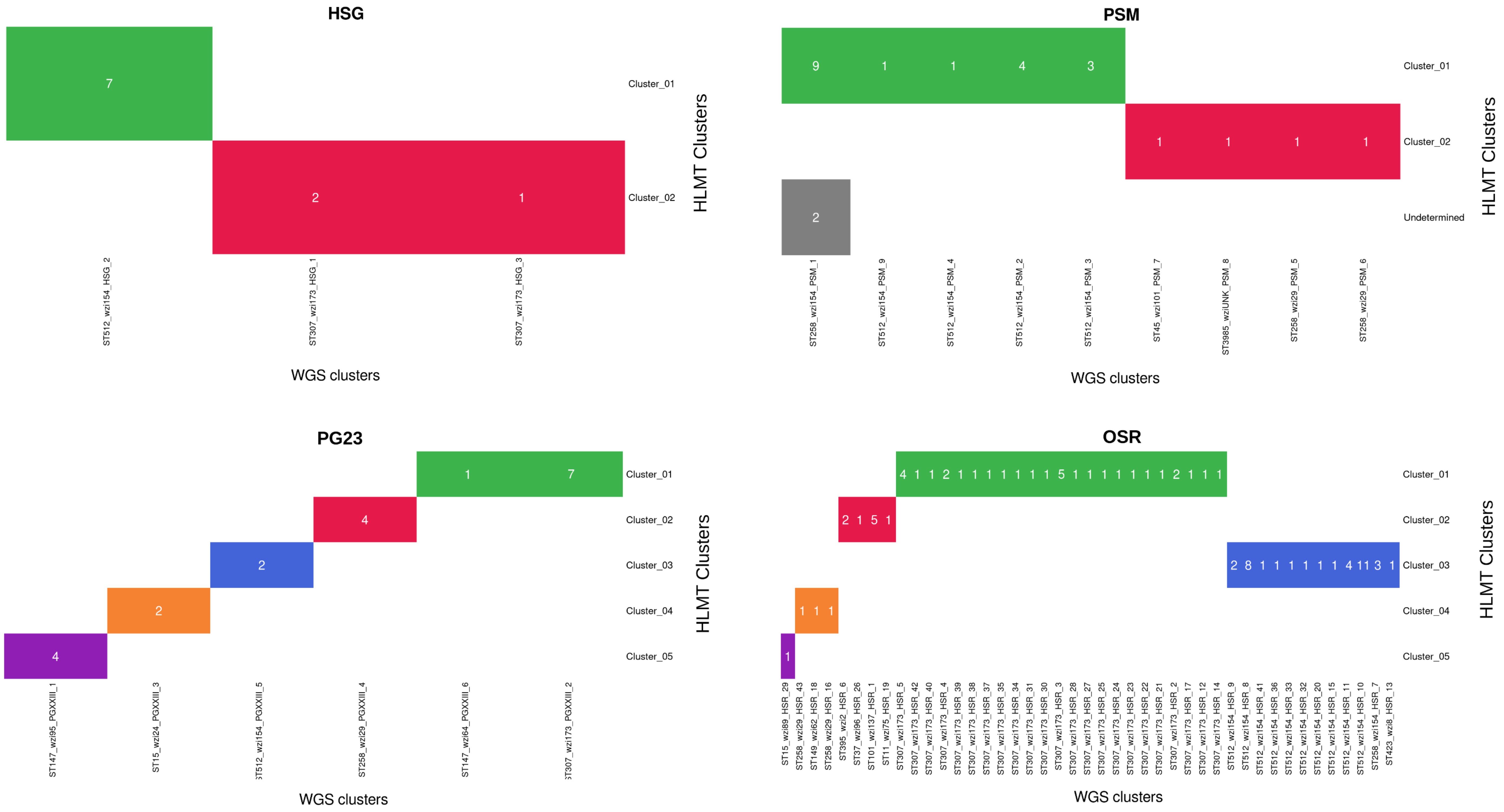

FigureS4: Contingency table of HLMT-clustering vs PFGE - For the OSR dataset, the HLMT clusters are compared to the PFGE clusters with a contingency table. Each color represents a HLMT cluster.

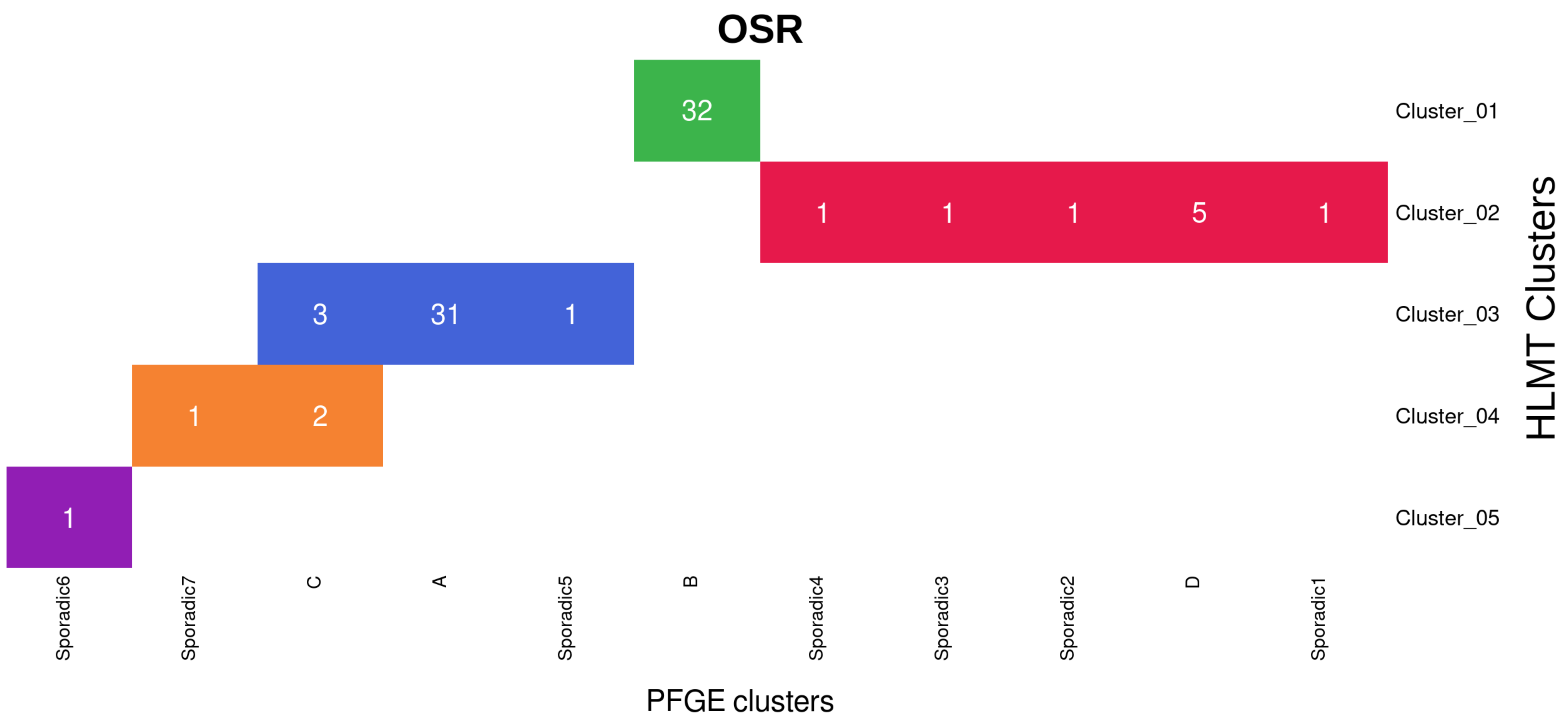

Table S1. Information about the strains included in the work: isolates metadata, genome features and typing results

ID_isolate	Metadata				Genome assemblies features							HRM experiments results						Typing results						
	Dataset	ID_patient	Date of isolation	Location	Assembly size	Contings	N50	Accession	Source	Used to build reference dataset (Pasala et. al 2020)	Used for HRM protocol development (Perini et. al 2020)	wzi-3			*wzi-4*			HLMT		MLST	*wzi*	K-locus	WGS Cluster	PFGE cluster
												T1	T2	T3	T1	T2	T3	HLMT-clustering	HLMT-assignment					
1753	PSM	p1	2015-06-02	ICU	5502862	74	231088	ERS3413479	Ferrari et al. 2019			84	84	84	84	84	83.5	Cluster_01	MT_ST258:512_wzi154	ST512	wzi154	KL107	PSM_8	
1758	PSM	p2	2015-06-23	ICU	5617895	76	203922	ERS3413480	Ferrari et al. 2019			84	84	84	84.5	84.5	84.5	Cluster_02	MT_ST258_wzi29	ST258	wzi29	KL106	PSM_9	
1760	PSM	p4	2015-06-29	ICU	5677735	103	270452	ERS3413481	Ferrari et al. 2019			84	84	84	85	84.5	85	Cluster_02	MT_ST258_wzi29	ST258	wzi29	KL106	PSM_4	
1826	PSM	p3	2015-08-11	ICU	5828867	107	182804	ERS3413482	Ferrari et al. 2019			85	85	85	84	84	84	Cluster_02	MT_ST11:101:15	ST45	wzi101	KL24	PSM_7	
1873	PSM	p5	2015-08-31	ICU	5723226	122	202463	ERS3413485	Ferrari et al. 2019			84	84	84	84	83.5	83.5	Cluster_01	MT_ST258:512_wzi154	ST512	wzi154	KL107	PSM_2	
1880	PSM	p6	2015-09-15	ICU	5725807	121	176884	ERS3413486	Ferrari et al. 2019			84	84	84	83.5	84	83.5	Cluster_01	MT_ST258:512_wzi154	ST512	wzi154	KL107	PSM_2	
1897	PSM	p7	2015-10-08	ICU	5716105	114	270452	ERS3413487	Ferrari et al. 2019			84	84	84	83.5	83.5	83.5	Cluster_01	MT_ST258:512_wzi154	ST512	wzi154	KL107	PSM_1	
1935	PSM	p8	2015-10-26	ICU	5712470	126	192161	ERS3413488	Ferrari et al. 2019			84	84	84	83.5	83.5	83.5	Cluster_01	MT_ST258:512_wzi154	ST512	wzi154	KL107	PSM_1	
1955	PSM	p9	2015-11-10	ICU	5715080	110	237759	ERS3413489	Ferrari et al. 2019			84	84	84	83.5	83.5	83.5	Cluster_01	MT_ST258:512_wzi154	ST512	wzi154	KL107	PSM_1	
1961	PSM	p10	2015-11-17	ICU	5715967	112	270452	ERS3413490	Ferrari et al. 2019			84	84	84	84	84	83.5	Cluster_01	MT_ST258:512_wzi154	ST512	wzi154	KL107	PSM_1	
1987	PSM	p11	2015-12-01	ICU	5629086	128	148646	ERS3413491	Ferrari et al. 2019			84	84	84	84	84	84	Undetermined	MT_ST258:512_wzi154	ST258	wzi154	KL107	PSM_3	
1998	PSM	p5	2015-12-01	ICU	5485760	106	174674	ERS3413492	Ferrari et al. 2019			84.5	84.5	84.5	84.5	84.5	84.5	Cluster_02	MT_ST10_wzi95	ST3985	wziUNK	KL151	PSM_6	
2003	PSM	p12	2015-12-08	ICU	5632071	110	202043	ERS3413493	Ferrari et al. 2019			84	84	84	84	83.5	83.5	Cluster_01	MT_ST258:512_wzi154	ST258	wzi154	KL107	PSM_3	
2079	PSM	p16	2016-10-26	ICU	5629997	104	231105	ERS3413496	Ferrari et al. 2019			84	84	84	83.5	83.5	83.5	Cluster_01	MT_ST258:512_wzi154	ST258	wzi154	KL107	PSM_3	
2106	PSM	p5	2016-02-02	ICU	5599789	162	154406	ERS3413497	Ferrari et al. 2019			84	84	84	83.5	83.5	83.5	Cluster_01	MT_ST258:512_wzi154	ST512	wzi154	KL107	PSM_2	
2110	PSM	p14	2016-02-02	ICU	5629281	127	157686	ERS3413498	Ferrari et al. 2019			84	84	84	83.5	83.5	83.5	Cluster_01	MT_ST258:512_wzi154	ST258	wzi154	KL107	PSM_3	
2133	PSM	p17	2016-02-16	ICU	5629136	112	203931	ERS3413499	Ferrari et al. 2019			84	84	84	83.5	83.5	83.5	Cluster_01	MT_ST258:512_wzi154	ST258	wzi154	KL107	PSM_3	
2165	PSM	p19	2016-03-01	ICU	5632771	105	176879	ERS3413501	Ferrari et al. 2019			84	84	84	83.5	83.5	83.5	Cluster_01	MT_ST258:512_wzi154	ST258	wzi154	KL107	PSM_3	
2176	PSM	p20	2016-03-08	ICU	5631696	108	202452	ERS3413503	Ferrari et al. 2019			84	84	84	83.5	83.5	83.5	Cluster_01	MT_ST258:512_wzi154	ST258	wzi154	KL107	PSM_3	
2182	PSM	p18	2016-03-15	ICU	5632328	107	186863	ERS3413504	Ferrari et al. 2019			84	84	84	83.5	83.5	83.5	Cluster_01	MT_ST258:512_wzi154	ST258	wzi154	KL107	PSM_3	
2183	PSM	p15	2016-03-15	ICU	5646608	126	213038	ERS3413505	Ferrari et al. 2019			84	84	84	83.5	83.5	83.5	Cluster_01	MT_ST258:512_wzi154	ST258	wzi154	KL107	PSM_3	
2205	PSM	p22	2016-04-04	ICU	5570370	116	166799	ERS3413507	Ferrari et al. 2019			84	84	84	83.5	83.5	83.5	Cluster_01	MT_ST258:512_wzi154	ST512	wzi154	KL107	PSM_5	
2218	PSM	p23	2016-04-19	ICU	5629856	106	203931	ERS3413508	Ferrari et al. 2019			84	84	84	84	84	83.5	Cluster_01	MT_ST258:512_wzi154	ST258	wzi154	KL107	PSM_3	
2221	PSM	p21	2016-04-19	ICU	5630079	105	202549	ERS3413509	Ferrari et al. 2019			84	84	84	84	84	84	Undetermined	MT_ST258:512_wzi154	ST258	wzi154	KL107	PSM_3	
BgKpn-1	PGXXIII	p1	2019-10-15	ICU adults 3	5526754	121	170413	ERR5908336	*This work*			84	84	84	82.5	82.5	82.5	Cluster_01	MT_ST307_wzi173---MT_ST147_wzi64	ST307	wzi173	KL102	PGXXIII_4	
BgKpn-10	PGXXIII	p8	2019-09-20	Heart failure 1 and heart transplant	5623572	120	270453	ERR5908337	*This work*			83.5	83.5	83.5	84.5	84.5	84.5	Cluster_02	MT_ST258_wzi29	ST258	wzi29	KL106	PGXXIII_2	
BgKpn-11	PGXXIII	p7	2019-08-16	Heart failure 1 and heart transplant	5628070	116	237760	ERR5908338	*This work*			84	84	83.5	83.5	83.5	83.5	Cluster_03	MT_ST258:512_wzi154	ST512	wzi154	KL107	PGXXIII_1	
BgKpn-12	PGXXIII	p9	2019-11-01	General surgery 3 and abdominal transplants	5554484	158	203622	ERR5908339	*This work*			84	84	84	82.5	82.5	82.5	Cluster_01	MT_ST307_wzi173---MT_ST147_wzi64	ST307	wzi173	KL102	PGXXIII_4	
BgKpn-13	PGXXIII	p9	2019-10-28	General surgery 3 and abdominal transplants	5530154	116	201727	ERR5908340	*This work*			84	84	84	82.5	82.5	82.5	Cluster_01	MT_ST307_wzi173---MT_ST147_wzi64	ST307	wzi173	KL102	PGXXIII_4	
BgKpn-14	PGXXIII	p9	2019-10-29	General surgery 3 and abdominal transplants	5541387	140	180695	ERR5908341	*This work*			84	84	84	82.5	82.5	82.5	Cluster_01	MT_ST307_wzi173---MT_ST147_wzi64	ST307	wzi173	KL102	PGXXIII_4	
BgKpn-15	PGXXIII	p10	2019-11-04	General surgery 3 and abdominal transplants	5528484	111	177836	ERR5908342	*This work*			84	84	84	82.5	82.5	82.5	Cluster_01	MT_ST307_wzi173---MT_ST147_wzi64	ST307	wzi173	KL102	PGXXIII_4	
BgKpn-16	PGXXIII	p11	2019-11-04	General surgery 3 and abdominal transplants	5528613	109	178006	ERR5908343	*This work*			84	84	84	82.5	82.5	82.5	Cluster_01	MT_ST307_wzi173---MT_ST147_wzi64	ST307	wzi173	KL102	PGXXIII_4	
BgKpn-17	PGXXIII	p12	2019-09-27	infectious diseases	5620531	116	247802	ERR5908344	*This work*			83.5	83.5	83.5	84.5	84.5	84.5	Cluster_02	MT_ST258_wzi29	ST258	wzi29	KL106	PGXXIII_2	
BgKpn-18	PGXXIII	p13	2019-08-01	Neurosurgery	5732580	169	181561	ERR5908345	*This work*			85	85	85	83.5	83.5	83.5	Cluster_04	MT_ST11:101:15	ST15	wzi24	KL24	PGXXIII_3	
BgKpn-2	PGXXIII	p1	2019-10-21	ICU adults 3	5546403	141	217083	ERR5908347	*This work*			84	84	84	82.5	82.5	82.5	Cluster_01	MT_ST307_wzi173---MT_ST147_wzi64	ST307	wzi173	KL102	PGXXIII_4	
BgKpn-20	PGXXIII	p15	2019-09-17	Coronary unit	5621782	115	270453	ERR5908346	*This work*			83.5	83.5	83.5	84.5	84.5	84.5	Cluster_02	MT_ST258_wzi29	ST258	wzi29	KL106	PGXXIII_2	
BgKpn-21	PGXXIII	p16	2019-09-17	Coronary unit	5620210	114	270453	ERR5908348	*This work*			83.5	83.5	83.5	84.5	84.5	84.5	Cluster_02	MT_ST258_wzi29	ST258	wzi29	KL106	PGXXIII_2	
BgKpn-3	PGXXIII	p2	2019-10-03	Heart surgery ICU	5816322	471	230462	ERR5908358	*This work*			85	85	85	84.5	84.5	84.5	Cluster_05	MT_ST10_wzi95	ST147	wzi95	KL20	PGXXIII_5	
BgKpn-4	PGXXIII	p3	2019-09-09	Heart surgery ICU	5580911	142	295825	ERR5908360	*This work*			85	85	85	84.5	84.5	84.5	Cluster_05	MT_ST10_wzi95	ST147	wzi95	KL20	PGXXIII_5	
BgKpn-5	PGXXIII	p4	2019-08-14	Neurosurgery ICU	5730527	163	198863	ERR5908361	*This work*			85	85	85	83.5	83.5	83.5	Cluster_04	MT_ST11:101:15	ST15	wzi24	KL24	PGXXIII_3	
BgKpn-6	PGXXIII	p3	2019-09-17	Neurosurgery ICU	5597170	136	311871	ERR5908362	*This work*			85	85	85	84.5	84.5	84.5	Cluster_05	MT_ST10_wzi95	ST147	wzi95	KL20	PGXXIII_5	
BgKpn-7	PGXXIII	p5	2019-10-08	Heart surgery 1	5644733	123	331088	ERR5908363	*This work*			85	85	85	84.5	84.5	84.5	Cluster_05	MT_ST10_wzi95	ST147	wzi95	KL20	PGXXIII_5	
BgKpn-8	PGXXIII	p6	2019-10-22	Heart surgery 1	5873033	118	295825	ERR5908364	*This work*			84	84	84	82	82	82	Cluster_01	MT_ST147_wzi64	ST147	wzi64	KL64	PGXXIII_6	
BgKpn-9	PGXXIII	p7	2019-08-12	Heart failure 1 and heart transplant	5622573	108	237760	ERR5908365	*This work*			83.5	83.5	83.5	83.5	83.5	83.5	Cluster_03	MT_ST258:512_wzi154	ST512	wzi154	KL107	PGXXIII_1	
KP10	HSR	p9	2017-02-16	General medicine	5601097	440	161089	SRX6820107	Gona et al. 2020			84.5	84.5	84	82.5	82.5	82.5	Cluster_01	MT_ST307_wzi173---MT_ST147_wzi64	ST307	wzi173	KL102	HSR_6	B
KP118	HSR	p18	2017-03-29	Transplants	5984794	325	101985	SRX6820113	Gona et al. 2020			84.5	84.5	84.5	84	84	84	Cluster_02	MT_ST11:101:15	ST395	wzi2	KL2	HSR_1	Sporadic4
KP119	HSR	p17	2017-03-27	Med.Endo-Met	5545418	306	160174	SRX6820112	Gona et al. 2020			84.5	84	84	82.5	82.5	82	Cluster_01	MT_ST307_wzi173---MT_ST147_wzi64	ST307	wzi173	KL102	HSR_18	B
KP12	HSR	p4	2017-01-31	Emergency room	6230596	2367	160177	SRX6820128	Gona et al. 2020			84	84	84	82.5	82.5	82.5	Cluster_01	MT_ST307_wzi173---MT_ST147_wzi64	ST307	wzi173	KL102	HSR_7	B
KP122	HSR	p21	2017-04-12	Emergency room	5573563	328	161149	SRX6820116	Gona et al. 2020			84	84	84	82.5	82.5	82	Cluster_01	MT_ST307_wzi173---MT_ST147_wzi64	ST307	wzi173	KL102	HSR_6	B
KP123	HSR	p20	2017-04-11	General surgery	5640957	395	129667	SRX6820115	Gona et al. 2020	x	x	83.5	83.5	83	83	83.5	83	Cluster_03	Unassigned	ST512	wzi154	KL107	HSR_8	A
KP124	HSR	p27	2017-05-02	Infectious diseases	5644756	337	204708	SRX6820122	Gona et al. 2020			84.5	84.5	84.5	83.5	83.5	83	Cluster_02	MT_ST11:101:15	ST101	wzi137	KL17	HSR_3	D
KP125	HSR	p29	2017-05-05	Medical laboratory	5531488	413	141155	SRX6820123	Gona et al. 2020			84	84	84	82	82.5	82	Cluster_01	MT_ST147_wzi64	ST307	wzi173	KL102	HSR_4	B
KP126	HSR	p31	2017-05-10	General medicine	5584279	290	129284	SRX6820127	Gona et al. 2020			84	84	84	82.5	82.5	82.5	Cluster_01	MT_ST307_wzi173---MT_ST147_wzi64	ST307	wzi173	KL102	HSR_6	B
KP127	HSR	p32	2017-05-10	Oncology	5718042	320	98700	SRX6820125	Gona et al. 2020			83.5	83.5	83.5	83.5	83.5	83	Cluster_03	MT_ST258:512_wzi154	ST512	wzi154	KL107	HSR_9	A
KP2	HSR	p10	2017-02-17	Emergency room	5579155	399	137838	SRX6820063	Gona et al. 2020			84.5	84.5	84.5	82.5	82.5	82.5	Cluster_01	MT_ST307_wzi173---MT_ST147_wzi64	ST307	wzi173	KL102	HSR_6	B
KP232	HSR	p36	2017-05-26	General surgery	5702818	234	108224	SRX6820131	Gona et al. 2020	x	x	83.5	83.5	83.5	83.5	83.5	83.5	Cluster_03	MT_ST258:512_wzi154	ST258	wzi154	KL107	HSR_11	C
KP234	HSR	p33	2017-05-10	Cardiac surgery	5509535	207	142001	SRX6820126	Gona et al. 2020			84	84	84	82.5	82.5	82.5	Cluster_01	MT_ST307_wzi173---MT_ST147_wzi64	ST307	wzi173	KL102	HSR_6	B
KP236	HSR	p25	2017-04-24	Neurology	5478361	248	137505	SRX6820120	Gona et al. 2020			84	84	84	82.5	82.5	82.5	Cluster_01	MT_ST307_wzi173---MT_ST147_wzi64	ST307	wzi173	KL102	HSR_7	B
KP238	HSR	p39	2017-06-09	Oncology	5567892	251	145553	SRX6820134	Gona et al. 2020			84	84	84	82.5	82.5	82.5	Cluster_01	MT_ST307_wzi173---MT_ST147_wzi64	ST307	wzi173	KL102	HSR_6	B
KP239	HSR	p38	2017-06-08	General medicine	5565938	281	124948	SRX6820133	Gona et al. 2020			83.5	83.5	83.5	83.5	83.5	83	Cluster_03	MT_ST258:512_wzi154	ST512	wzi154	KL107	HSR_2	A
KP240	HSR	p40	2017-06-09	Emergency room	5571853	340	108845	SRX6820135	Gona et al. 2020			84	84	84	82.5	82.5	82.5	Cluster_01	MT_ST307_wzi173---MT_ST147_wzi64	ST307	wzi173	KL102	HSR_6	B
KP242	HSR	p13	2017-02-24	Oncology	5491601	272	99162	SRX6820109	Gona et al. 2020			84	84	84	83.5	83.5	83.5	Cluster_03	MT_ST258:512_wzi154	ST512	wzi154	KL107	HSR_10	A
KP243	HSR	p14	2017-03-03	Cardiac surgery	5490806	203	142332	SRX6820110	Gona et al. 2020			84	84	84	82.5	82.5	82.5	Cluster_01	MT_ST307_wzi173---MT_ST147_wzi64	ST307	wzi173	KL102	HSR_6	B
KP245	HSR	p23	2017-04-15	Urgencies	5516541	243	141155	SRX6820118	Gona et al. 2020			84	84	84	82.5	82.5	82.5	Cluster_01	MT_ST307_wzi173---MT_ST147_wzi64	ST307	wzi173	KL102	HSR_6	B
KP246	HSR	p28	2017-05-03	Pancreas surgery	5534322	238	128623	SRX6820130	Gona et al. 2020	x	x	83.5	83.5	83.5	84.5	84.5	84.5	Cluster_04	MT_ST258_wzi29	ST258	wzi29	KL106	HSR_15	C
KP248	HSR	p35	2017-05-16	Neurology	5686959	407	86320	SRX6820129	Gona et al. 2020		x	84	83.5	83.5	83.5	83.5	83.5	Cluster_03	MT_ST258:512_wzi154	ST423	wzi8	KL8	HSR_16	Sporadic5
KP249	HSR	p41	2017-07-03	General surgery	5564049	340	87671	SRX6820137	Gona et al. 2020			83.5	83.5	83.5	83.5	83.5	83	Cluster_03	MT_ST258:512_wzi154	ST512	wzi154	KL107	HSR_2	A
KP251	HSR	p42	2017-07-11	Oncology	5590376	192	139326	SRX6820138	Gona et al. 2020			84	84	84	82.5	82.5	82.5	Cluster_01	MT_ST307_wzi173---MT_ST147_wzi64	ST307	wzi173	KL102	HSR_7	B
KP252	HSR	p44	2017-07-14	General surgery	5708317	268	100647	SRX6820065	Gona et al. 2020	x	x	83.5	83.5	83.5	83.5	83.5	83.5	Cluster_03	MT_ST258:512_wzi154	ST258	wzi154	KL107	HSR_11	C
KP255	HSR	p52	2017-08-12	Neurology	5664099	209	134991	SRX6820072	Gona et al. 2020			85	85	85	83.5	83.5	83.5	Cluster_02	MT_ST11:101:15	ST101	wzi137	KL17	HSR_3	D
KP256	HSR	p48	2017-07-25	General medicine	5568136	316	96282	SRX6820069	Gona et al. 2020			83.5	83.5	83.5	83.5	83.5	83	Cluster_03	MT_ST258:512_wzi154	ST512	wzi154	KL107	HSR_2	A
KP257	HSR	p49	2017-07-28	Med.Endo-Met	5632827	282	99046	SRX6820070	Gona et al. 2020			83.5	83.5	83.5	83.5	83.5	83	Cluster_03	MT_ST258:512_wzi154	ST512	wzi154	KL107	HSR_8	A
KP258	HSR	p24	2017-04-19	Cardiac surgery	5569727	283	135968	SRX6820119	Gona et al. 2020			84	84	84	82.5	82.5	82.5	Cluster_01	MT_ST307_wzi173---MT_ST147_wzi64	ST307	wzi173	KL102	HSR_6	B
KP260	HSR	p54	2017-09-08	General surgery	5671346	248	106510	SRX6820075	Gona et al. 2020			83.5	83.5	83.5	83.5	83	83	Cluster_03	Unassigned	ST512	wzi154	KL107	HSR_10	A
KP261	HSR	p47	2017-07-24	General medicine	5530739	223	131661	SRX6820068	Gona et al. 2020	x	x	83.5	83.5	83.5	84.5	84.5	84	Cluster_04	MT_ST258_wzi29	ST258	wzi29	KL106	HSR_13	C
KP265	HSR	p45	2017-07-18	Urology	5494327	372	177478	SRX6820066	Gona et al. 2020			84	84	84	82.5	82.5	82	Cluster_01	MT_ST307_wzi173---MT_ST147_wzi64	ST307	wzi173	KL102	HSR_14	B
KP355	HSR	p56	2017-09-18	Cardiac surgery	5485862	237	132212	SRX6820076	Gona et al. 2020			84	83.5	83.5	83.5	83.5	83.5	Cluster_03	MT_ST258:512_wzi154	ST512	wzi154	KL107	HSR_2	A
KP356	HSR	p55	2017-09-15	Oncology	5300651	179	177478	SRX6820073	Gona et al. 2020			84	84	84	82.5	82.5	82.5	Cluster_01	MT_ST307_wzi173---MT_ST147_wzi64	ST307	wzi173	KL102	HSR_4	B
KP359	HSR	p57	2017-09-26	General medicine	5733367	215	172705	SRX6820077	Gona et al. 2020	x	x	83.5	84	83.5	84	84	84	Cluster_04	MT_ST258:512_wzi154	ST441	wzi149	KL62	HSR_20	Sporadic7
KP360	HSR	p60	2017-10-04	Transplants	5562359	260	137581	SRX6820080	Gona et al. 2020			83.5	83.5	83.5	83.5	83.5	83.5	Cluster_03	MT_ST258:512_wzi154	ST512	wzi154	KL107	HSR_2	A
KP361	HSR	p62	2017-10-09	General surgery	5703868	350	104371	SRX6820084	Gona et al. 2020			84	84	84	83.5	83.5	83.5	Cluster_03	MT_ST258:512_wzi154	ST512	wzi154	KL107	HSR_8	A

Table S1. Information about the strains included in the work: isolates metadata, genome features and typing results

ID_isolate	Metadata				Genome assemblies features							HRM experiments results						Typing results						
	Dataset	ID_patient	Date of isolation	Location	Assembly size	Contings	N50	Accession	Source	Used to build reference dataset (Pasala et. al 2020)	Used for HRM protocol development (Perini et. al 2020)	wzi-3			*wzi-4*			HLMT		MLST	*wzi*	K-locus	WGS Cluster	PFGE cluster
												T1	T2	T3	T1	T2	T3	HLMT-clustering	HLMT-assignment					
KP363	HSR	p59	2017-10-02	General surgery	5705965	342	128848	SRX6820079	Gona et al. 2020			83.5	83.5	83.5	83	83	83	Cluster_03	Unassigned	ST512	wzi154	KL107	HSR_8	A
KP364	HSR	p61	2017-10-06	General surgery	5708394	401	100646	SRX6820081	Gona et al. 2020			83.5	83.5	83.5	83	83	83	Cluster_03	Unassigned	ST512	wzi154	KL107	HSR_8	A
KP365	HSR	p63	2017-10-12	Neurology	5660865	208	152507	SRX6820083	Gona et al. 2020			85	85	84.5	83.5	83.5	83.5	Cluster_02	MT_ST11:101:15	ST101	wzi137	KL17	HSR_3	D
KP366	HSR	p2	2017-01-23	Hematology	5588839	251	108880	SRX6820117	Gona et al. 2020	x	x	85	85	84.5	83.5	83.5	83.5	Cluster_02	MT_ST11:101:15	ST11	wzi75	KL105	HSR_12	Sporadic2
KP367	HSR	p66	2017-10-25	Transplants	5566489	285	137355	SRX6820087	Gona et al. 2020			83.5	83.5	83.5	83.5	83	83	Cluster_03	Unassigned	ST512	wzi154	KL107	HSR_2	A
KP368	HSR	p68	2017-11-03	Infectious diseases	5553507	280	109634	SRX6820089	Gona et al. 2020			83.5	83.5	83	83.5	83.5	83	Cluster_03	MT_ST258:512_wzi154	ST512	wzi154	KL107	HSR_21	A
KP4	HSR	p5	2017-02-06	Cardiac surgery	5582774	314	179295	SRX6820139	Gona et al. 2020			84	84	84	82.5	82.5	82.5	Cluster_01	MT_ST307_wzi173---MT_ST147_wzi64	ST307	wzi173	KL102	HSR_6	B
KP468	HSR	p1	2017-01-10	General surgery	5663210	309	73852	SRX6820061	Gona et al. 2020			83.5	83.5	83.5	83.5	83.5	83	Cluster_03	MT_ST258:512_wzi154	ST512	wzi154	KL107	HSR_8	A
KP469	HSR	p3	2017-01-23	Urology	5982949	322	90141	SRX6820062	Gona et al. 2020		x	84.5	84.5	84.5	83.5	83.5	83	Cluster_02	MT_ST11:101:15	ST395	wzi2	KL2	HSR_1	Sporadic1
KP471	HSR	p6	2017-02-06	General medicine	5520286	215	108882	SRX6820074	Gona et al. 2020			84	84	84	82.5	82	82	Cluster_01	MT_ST147_wzi64	ST307	wzi173	KL102	HSR_6	B
KP472	HSR	p7	2017-02-11	Cardiac surgery	5517715	241	141170	SRX6820085	Gona et al. 2020			84	84	84	82.5	82.5	82	Cluster_01	MT_ST307_wzi173---MT_ST147_wzi64	ST307	wzi173	KL102	HSR_6	B
KP473	HSR	p8	2017-02-13	Pancreas surgery	5956538	222	134991	SRX6820096	Gona et al. 2020		x	85	85	85	83.5	83.5	83.5	Cluster_02	MT_ST11:101:15	ST101	wzi137	KL17	HSR_3	D
KP475	HSR	p11	2017-02-17	Neurology	5599328	235	99560	SRX6820108	Gona et al. 2020			84	84	84	82.5	82	82	Cluster_01	MT_ST147_wzi64	ST307	wzi173	KL102	HSR_7	B
KP476	HSR	p15	2017-03-09	Cardiac surgery	5601647	259	150539	SRX6820111	Gona et al. 2020			84	84	83.5	82	82	82	Cluster_01	MT_ST147_wzi64	ST307	wzi173	KL102	HSR_6	B
KP477	HSR	p16	2017-03-09	Pancreas surgery	5327927	313	74554	SRX6820103	Gona et al. 2020			85	85	85	84.5	84.5	84.5	Cluster_02	MT_ST10_wzi95	ST37	wzi96	KL38	HSR_19	Sporadic3
KP478	HSR	p19	2017-04-04	Infectious diseases	5630417	329	89461	SRX6820114	Gona et al. 2020			83.5	83.5	83	83	83	83	Cluster_03	Unassigned	ST512	wzi154	KL107	HSR_8	A
KP479	HSR	p22	2017-04-14	Urology	5397504	214	129655	SRX6820104	Gona et al. 2020			84	84	84	82.5	82.5	82	Cluster_01	MT_ST307_wzi173---MT_ST147_wzi64	ST307	wzi173	KL102	HSR_7	B
KP480	HSR	p26	2017-04-29	General medicine	5567514	277	141155	SRX6820121	Gona et al. 2020			84	84	84	82.5	82	82	Cluster_01	MT_ST147_wzi64	ST307	wzi173	KL102	HSR_6	B
KP481	HSR	p30	2017-05-09	Cardiac surgery	5564651	341	105593	SRX6820124	Gona et al. 2020			84	84	84	82.5	82.5	82	Cluster_01	MT_ST307_wzi173---MT_ST147_wzi64	ST307	wzi173	KL102	HSR_6	B
KP485	HSR	p34	2017-05-15	Hematology	5967692	290	109315	SRX6820105	Gona et al. 2020	x	x	82	82	81.5	83.5	83	83	Cluster_05	Unassigned	ST15	wzi89	KL110	HSR_17	Sporadic6
KP486	HSR	p37	2017-06-05	Cardiac surgery	5563692	357	100396	SRX6820132	Gona et al. 2020			84	84	84	82.5	82.5	82	Cluster_01	MT_ST307_wzi173---MT_ST147_wzi64	ST307	wzi173	KL102	HSR_6	B
KP488	HSR	p50	2017-08-01	Transplants	5701430	269	100647	SRX6820136	Gona et al. 2020	x	x	83.5	83.5	83.5	83.5	83.5	83.5	Cluster_03	MT_ST258:512_wzi154	ST258	wzi154	KL107	HSR_11	C
KP489	HSR	p43	2017-07-11	Pancreas surgery	5493095	208	123755	SRX6820140	Gona et al. 2020			83.5	83.5	83.5	83	83	83	Cluster_03	Unassigned	ST512	wzi154	KL107	HSR_5	A
KP491	HSR	p46	2017-07-20	General surgery	5563329	292	105786	SRX6820067	Gona et al. 2020			84	84	84	82.5	82.5	82	Cluster_01	MT_ST307_wzi173---MT_ST147_wzi64	ST307	wzi173	KL102	HSR_6	B
KP492	HSR	p51	2017-08-02	Med.Endo-Met	5729741	410	87773	SRX6820071	Gona et al. 2020			83.5	83.5	83.5	83	83	83	Cluster_03	Unassigned	ST512	wzi154	KL107	HSR_22	A
KP493	HSR	p53	2017-08-18	Hematology	5765489	262	109111	SRX6820106	Gona et al. 2020			84	84	84	82.5	82.5	82	Cluster_01	MT_ST307_wzi173---MT_ST147_wzi64	ST307	wzi173	KL102	HSR_6	B
KP496	HSR	p58	2017-09-28	Cardiac surgery	5568843	280	129667	SRX6820078	Gona et al. 2020			83.5	83.5	83.5	83.5	83.5	83	Cluster_03	MT_ST258:512_wzi154	ST512	wzi154	KL107	HSR_2	A
KP498	HSR	p64	2017-10-12	Pancreas surgery	8411344	6365	59511	SRX6820082	Gona et al. 2020			83.5	83.5	83.5	83.5	83	83	Cluster_03	Unassigned	ST512	wzi154	KL107	HSR_5	A
KP499	HSR	p65	2017-10-17	Infectious diseases	5714750	364	126325	SRX6820086	Gona et al. 2020			83.5	83.5	83.5	83.5	83.5	83.5	Cluster_03	MT_ST258:512_wzi154	ST512	wzi154	KL107	HSR_10	A
KP501	HSR	p69	2017-11-23	Neurology	5724639	364	126256	SRX6820090	Gona et al. 2020			83.5	83.5	83	83	83	83	Cluster_03	Unassigned	ST512	wzi154	KL107	HSR_9	A
KP502	HSR	p70	2017-12-09	Cardiac surgery	5562596	385	100646	SRX6820091	Gona et al. 2020			83.5	83.5	83.5	83	83	83	Cluster_03	Unassigned	ST512	wzi154	KL107	HSR_2	A
KP596	HSR	p72	2017-12-15	General medicine	5519782	241	145379	SRX6820092	Gona et al. 2020			84	84	84	82.5	82.5	82	Cluster_01	MT_ST307_wzi173---MT_ST147_wzi64	ST307	wzi173	KL102	HSR_6	B
KP597	HSR	p73	2017-12-15	Neurology	5516754	224	133441	SRX6820093	Gona et al. 2020			85	85	84.5	83.5	83.5	83.5	Cluster_02	MT_ST11:101:15	ST101	wzi137	KL17	HSR_3	D
KP598	HSR	p76	2017-12-21	General medicine	5706003	346	126336	SRX6820097	Gona et al. 2020			83.5	83.5	83.5	83.5	83.5	83.5	Cluster_03	MT_ST258:512_wzi154	ST512	wzi154	KL107	HSR_8	A
KP599	HSR	p74	2017-12-18	Hematology	5746185	258	141155	SRX6820094	Gona et al. 2020			84	84	84	82.5	82.5	82.5	Cluster_01	MT_ST307_wzi173---MT_ST147_wzi64	ST307	wzi173	KL102	HSR_6	B
KP601	HSR	p75	2017-12-20	Cardiac surgery	5642547	333	106510	SRX6820095	Gona et al. 2020			83.5	83.5	83.5	83.5	83.5	83	Cluster_03	MT_ST258:512_wzi154	ST512	wzi154	KL107	HSR_23	A
KP603	HSR	p71	2017-12-13	General medicine	5646735	301	117369	SRX6820098	Gona et al. 2020			83.5	83.5	83.5	83.5	83.5	83	Cluster_03	MT_ST258:512_wzi154	ST512	wzi154	KL107	HSR_9	A
KP604	HSR	p77	2017-12-27	General surgery	5565100	291	109629	SRX6820099	Gona et al. 2020			83.5	83.5	83.5	83.5	83	83	Cluster_03	Unassigned	ST512	wzi154	KL107	HSR_2	A
KP605	HSR	p78	2017-12-27	Neurology	5565093	284	111064	SRX6820101	Gona et al. 2020			83.5	83.5	83.5	83	83	83	Cluster_03	Unassigned	ST512	wzi154	KL107	HSR_2	A
KP606	HSR	p79	2017-12-30	Neurology	5614662	284	99590	SRX6820102	Gona et al. 2020			84	84	84	82.5	82.5	82	Cluster_01	MT_ST307_wzi173---MT_ST147_wzi64	ST307	wzi173	KL102	HSR_6	B
KP613	HSR	p67	2017-10-30	General surgery	5508422	280	119064	SRX6820088	Gona et al. 2020			84	84	84	82.5	82.5	82.5	Cluster_01	MT_ST307_wzi173---MT_ST147_wzi64	ST307	wzi173	KL102	HSR_6	B
KP715	HSR	p80	2017-12-30	General surgery	5573004	304	115718	SRX6820100	Gona et al. 2020			83.5	83.5	83.5	83.5	83.5	83.5	Cluster_03	MT_ST258:512_wzi154	ST512	wzi154	KL107	HSR_2	A
KP9	HSR	p12	2017-02-21	Transplants	5692586	344	128796	SRX6820064	Gona et al. 2020			83.5	83.5	83.5	83.5	83.5	83	Cluster_03	MT_ST258:512_wzi154	ST512	wzi154	KL107	HSR_9	A
MZ438	HSG	p1	2018-08-14	Oncohematology	5695324	147	202450	ERR5908349	*This work*			83.5	83.5	83.5	83.5	83.5	83.5	Cluster_01	MT_ST258:512_wzi154	ST512	wzi154	KL107	HSG_2	
MZ439	HSG	p2	2018-08-17	Oncohematology	5794075	111	204450	ERR5908350	*This work*			84	84	84	82.5	82.5	82.5	Cluster_02	MT_ST307_wzi173---MT_ST147_wzi64	ST307	wzi173	KL102	HSG_1	
MZ440	HSG	p3	2018-08-20	Oncohematology	5695054	150	205856	ERR5908351	*This work*			83.5	83.5	83.5	83.5	83.5	83.5	Cluster_01	MT_ST258:512_wzi154	ST512	wzi154	KL107	HSG_2	
MZ441	HSG	p4	2018-08-21	Oncohematology	5693386	148	202463	ERR5908352	*This work*			83.5	83.5	83.5	83.5	83.5	83.5	Cluster_01	MT_ST258:512_wzi154	ST512	wzi154	KL107	HSG_2	
MZ445	HSG	p5	2018-08-27	Oncohematology	5694930	148	203938	ERR5908353	*This work*			83.5	83.5	83.5	83.5	83.5	83.5	Cluster_01	MT_ST258:512_wzi154	ST512	wzi154	KL107	HSG_2	
MZ449	HSG	p6	2018-09-03	Oncohematology	5695402	145	219210	ERR5908354	*This work*			83.5	83.5	83.5	83.5	83.5	83.5	Cluster_01	MT_ST258:512_wzi154	ST512	wzi154	KL107	HSG_2	
MZ452	HSG	p7	2018-09-06	Oncohematology	5713244	180	205856	ERR5908355	*This work*			83.5	83.5	83.5	83.5	83.5	83.5	Cluster_01	MT_ST258:512_wzi154	ST512	wzi154	KL107	HSG_2	
MZ457	HSG	p8	2018-09-17	Oncohematology	5579924	113	204450	ERR5908356	*This work*			84	84	84	82.5	82.5	82.5	Cluster_02	MT_ST307_wzi173---MT_ST147_wzi64	ST307	wzi173	KL102	HSG_1	
MZ458	HSG	p9	2018-09-19	Oncohematology	5701303	157	203938	ERR5908357	*This work*			83.5	83.5	83.5	83.5	83.5	83.5	Cluster_01	MT_ST258:512_wzi154	ST512	wzi154	KL107	HSG_2	
MZ463	HSG	p10	2018-09-24	Oncohematology	5577394	109	315799	ERR5908359	*This work*			84	84	84	82.5	82.5	82.5	Cluster_02	MT_ST307_wzi173---MT_ST147_wzi64	ST307	wzi173	KL102	HSG_1	
